# PIK3CA-CDKN2A clonal evolution in metastatic breast cancer and multiple points cell-free DNA analysis

**DOI:** 10.1186/s12935-019-0991-y

**Published:** 2019-10-28

**Authors:** Maria Palmieri, Margherita Baldassarri, Francesca Fava, Alessandra Fabbiani, Giuseppe Maria Campennì, Maria Antonietta Mencarelli, Rossella Tita, Stefania Marsili, Alessandra Renieri, Elisa Frullanti

**Affiliations:** 1 Medical Genetics Unit, Policlinico “Santa Maria alle Scotte”, University of Siena, Viale Bracci, 2, 53100 Siena, Italy; 2 Genetica Medica, Azienda Ospedaliera Universitaria Senese, Siena, Italy; 3 European Institute of Oncology, Milan, Italy; 4 Oncology, Azienda Ospedaliera Universitaria Senese, Siena, Italy

**Keywords:** PIK3CA-CDKN2A, cfDNA, Liquid biopsy, Deep-next generation sequencing, Targeted-therapy

## Abstract

**Background:**

Daily experience tells us that breast cancer can be controlled using standard protocols up to the advent of a relapse. Now new frontiers in precision medicine like liquid biopsy of cell free DNA (cfDNA) give us the possibility to understand cancer evolution and pick up the key mutation on specific cancer driver gene. However, tight schedule of standardized protocol may impair the use of personalized experimental drugs in a timely therapeutic window.

**Main body:**

Here, using a combination of deep next generation sequencing and cfDNA liquid biopsy, we demonstrated that it is possible to monitor cancer relapse over time. We showed for the first time the exact correspondence from the increasing clonal expansion and clinical worsening of metastatic breast cancer.

**Conclusion:**

Thanks to liquid biopsy may be possible to introduce new experimental drugs in the correct therapeutic window which would lead in the near future to an effective treatment which otherwise remains challenging.

## Background

Melchardt et al. 2018 demonstrated that clones in distant relapse of head and neck cancer are different in respect to those identified at the beginning in tumor biopsy [1]. Classically, haematological malignancies have taught us that, within the dynamic clonal evolution, a subclonal expansion of a pre-existing mutated clone leads often to relapse [2, 3]. Expanding clones may be selected by treatment acquiring drug resistance and patients who relapse after an effective therapy usually have a poor prognosis [4, 5]. While at the beginning of tumor expansion there is a consistent, although variable, mutational burden from ten to hundred clones, at relapse the leading clone is usually only one [5]. Liquid biopsy of cell free DNA (cfDNA) has now the potential to follow the temporal evolution and to inform us about the driver mutation of the expanded clone. Among solid tumors, breast cancer is one of the most facing a high risk of recurrence after curative surgery and therapeutic treatment. *PIK3CA* mutations are found in 27% of cases of disease progression in breast cancer [6]. Free survival was demonstrated to be inversely correlated with *PIK3CA* mutations at liquid biopsy [7] but longitudinal analysis of clonal evolution and disease progression was missed.

## Main text

Using a combination of deep next generation sequencing and cfDNA liquid biopsy (Oncomine pan-cancer cell free assay and tissues/blood custom panels on ion proton platform, life technologies), we showed for the first time the exact correspondence between the increasing clonal expansion and the clinical worsening of metastatic breast cancer in a 44-year-old female with disease recurrence after 4 years and half of disease control. At 38 years she presented a poorly differentiated ductal carcinoma of the right breast, pT1cN1mi(1/3) G3, estrogen receptor (ER) positive 90%, progesterone receptor (PgR) positive 40%, HER2 negative, KI67 40%. She underwent quadrantectomy and subsequent chemotherapy with Epirubicin Ciclofosfamide (EC) for four cycles and Decapeptyl every 28 days. After 7 months, she added tamoxifen for 1 year, then replaced by Exemestrane for 2 years. Radiotherapy was also performed. Family history was positive for breast cancer in the paternal side although exome analysis failed to reveal germline mutation in known cancer driver genes.

After 4 years and half from the diagnosis, liver and bone metastasis were detected. Biopsy of the VIII hepatic segment showed metastasis of breast carcinoma, ER negative, PgR negative, HER2 negative, Ki67 17%. Subsequently, therapy with Paclitaxel was undertaken weekly. Three months later from the previous evidence of metastasis, numerical and dimensional increase in liver lesions was demonstrated. Monotherapy with cisplatin was started and then in association with capecitabine and epirubicin (ECX scheme) with dose of 80% for seven consecutive cycles. Afterwards, she had treatment with Capecitabine and metronomic cyclophosphamide for 3 months, then three cycles of eribuline were performed. Therapy with carboplatin and gemcitabine was subsequently administered. One month later, since there was an increase of liver enzymes, monotherapy with carboplatin was undertaken. No other treatment options were available given the disease progression.

**Fig. 1:**
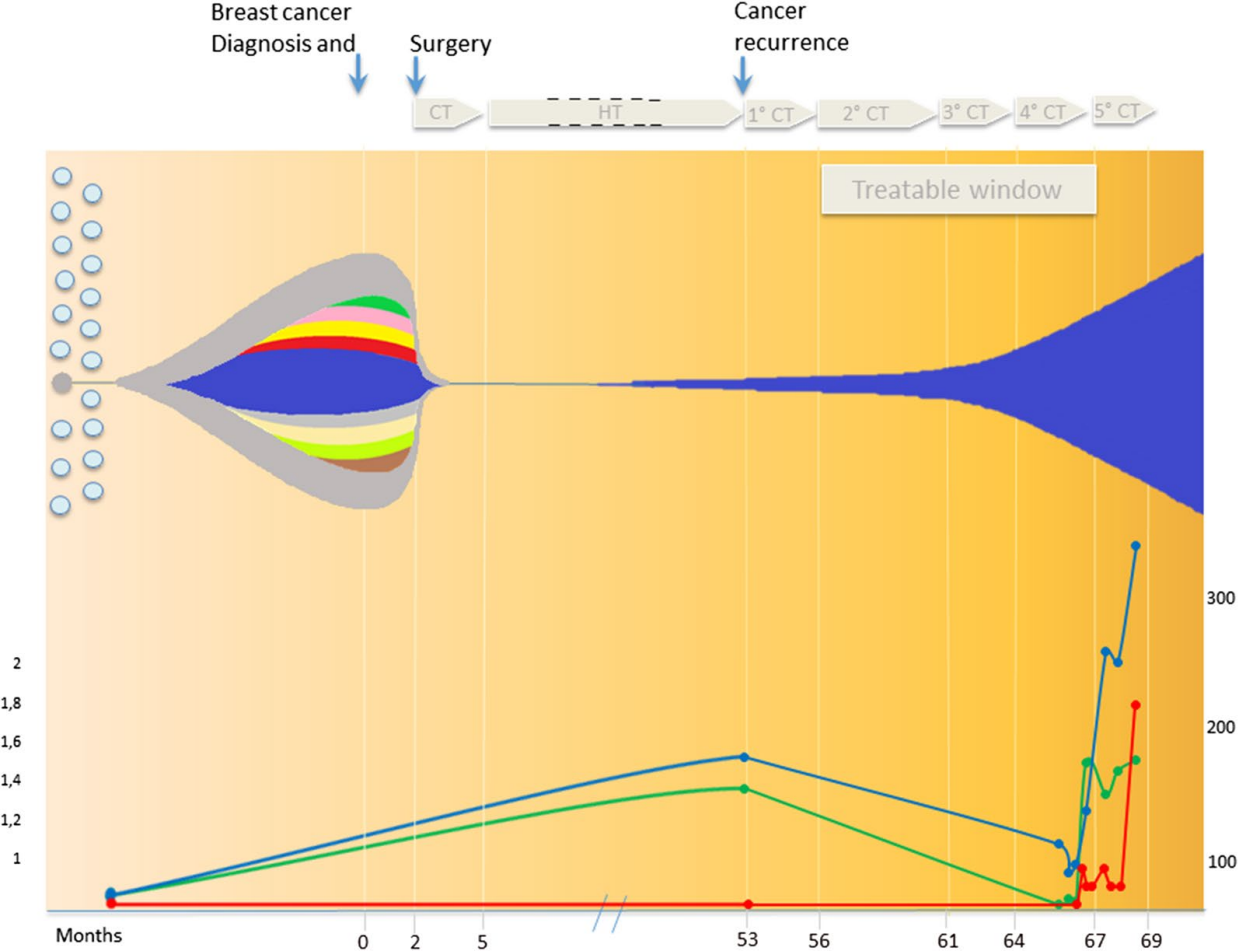
Outline of clonal and subclonal architecture and clinical outcome. Upper panel: fish plot of clonal evolution and timing. At the primary tumor biopsy, 10 different distinctive clones with different mutational load (ranging from 0.3 to 25%) were identified: IDH1 (green), CTNNB1 (pink), FBXW7 (yellow), APC (red), EGFR (beige), PIK3CA (blue), PTEN (brown), SMAD4 (grey), CDKN2A (blu), GNAS (light green). Among these, the clone-bearing PIK3CA/CDKN2A mutations (blue) increases from 25% at the time of primary tumor biopsy to around 50% at 180 days’ post-recurrence. Bottom panel: liver enzymes (AST = blue line; ALT = green line; values in U/L on the right) and bilirubin (red line; values in mg/dL on the left) whose values increase according to disease progression. *CT* chemotherapy; *HT* hormone therapy

After about 1 year from the evidence of bones and liver metastasis, we performed a first cfDNA analysis, which revealed pathogenic mutations in *PIK3CA* gene [c.1633G>A; p.(Glu545Lys)] and *CDKN2A* [c.1904T>G; p.(Leu635Arg)]. Five months later, a second cfDNA analysis highlighted an exponential increase of clones with the same pathogenic mutations (Fig. 1). In comparison with the mutational burden identified at primary tumor biopsy, the expanding clone has a simplified architecture (Fig. 1).

We demonstrated here that multiple points cfDNA analysis give now the possibility to understand the overall cancer dynamics and pick up the key mutation leading to cancer recurrence, separating them from not expanding occasional clones. Large comprehensive analysis of haematological malignancies indicates a complex temporal dynamic landing although in few (from one to six) driver mutations at late relapse [8]. Likewise, in breast cancer one (or few) is the driver mutation leading to cancer recurrence and it is of pivotal importance to target this driver mutation(s) on time. However, stick too tightly to standardised protocol may impair the use of personalised experimental drugs in a timely therapeutic window.

Data reported here indicate that there is a tight therapeutic window useful for counteract final clonal expansion and that the minimally invasive cfDNA analysis allows a close and dynamic monitoring of clonal evolution. This is also supported by the mathematical model developed by Khan et al. [9]. In our case, an innovative and more effective therapy could be CDK4/6 inhibitors in combination with PI3 K-specific inhibitor initiated at the beginning of the clonal expansion [10]. Introducing of experimental drug in the correct therapeutic window would lead in the near future to effective treatment which otherwise remain challenging.

## Conclusions

In conclusion, we demonstrated that multiple points cfDNA analysis reflects clonal evolution and allows track the evolving molecular landscapes of growing cancer cells by capturing broader molecular alterations that could hinder targeted treatments efficacy. The shorter turnaround time of cfDNA analysis and its high sensitivity and specificity are key factors to provide novel opportunities for adaptive personalised therapies, optimizing healthcare resources and enabling higher treatment efficacy and lower side-effects.

## Data Availability

The datasets used and/or analyzed during the current study are available from the corresponding author on reasonable request.

## Abbreviations

CDKN2A: cyclin dependent kinase inhibitor 2A
CDK: cyclin dependent kinase
cfDNA: cell-free DNA
EC: epirubicin ciclofosfamide
ECX: epirubicin cisplatin and capecitabine
ER: estrogen receptors
HER2: human epidermal growth factor receptor 2
PgR: progesterone receptor
PIK3CA: phosphoinositide-3-kinase, catalytic, alpha polypeptide
